# Laser Ablation Remote-Electrospray Ionisation Mass Spectrometry (LARESI MSI) Imaging—New Method for Detection and Spatial Localization of Metabolites and Mycotoxins Produced by Moulds

**DOI:** 10.3390/toxins12110720

**Published:** 2020-11-18

**Authors:** Justyna Szulc, Tomasz Ruman

**Affiliations:** 1 Department of Environmental Biotechnology, Lodz University of Technology, Wólczańska 171/173, 90-924 Łódź, Poland; 2 Faculty of Chemistry, Rzeszów University of Technology, 6 Powstańców Warszawy Ave., 35-959 Rzeszów, Poland; tomruman@prz.edu.pl

**Keywords:** mass spectrometry imagining, laser ablation, desorption/ionization mass spectrometry metabolites, metabolomic analysis, mycotoxins

## Abstract

To date, no method has been developed to assess the distribution of mycotoxins on the surface of grains, or other plant material, and the depth of their penetration into the interior. The Infrared (IR) Laser Ablation-Remote-Electrospray Ionization (LARESI) platform coupled to a tandem mass spectrometer (MS/MS), measuring in selected reaction monitoring (SRM) mode, was employed for the targeted imaging of selected metabolites of *Aspergillus fumigatus*, including mycotoxins in biological objects for the first time. This methodology allowed for the localisation of grain metabolites and fungal metabolites of grain infected by this mould. The distribution of metabolites in spelt grain was differentiated: fumigaclavine C, fumitremorgin C, and fumiquinazoline D were located mainly in the embryo, brevianamide F in the seed coat, and fumagillin in the endosperm. The LARESI mass spectrometry imaging method can be used in the future for the metabolomic analysis of mould metabolites in various plants and agricultural products.

## 1. Introduction

Moulds produce a wide variety of primary and secondary metabolites, including mycotoxins. They comprise approximately 400 low-molecular-weight substances, which exhibit great structural diversity and thermal and chemical stability [1,2]. Mycotoxins are produced by more than 200 identified moulds, belonging primarily to the genera *Aspergillus, Fusarium,* and *Penicillium* [3,4,5].

The presence of mould secondary metabolites in food, beverages, and feed has been recognised as a potential threat to human and animal health. Acute and chronic diseases can arise, caused mainly by the direct contamination of plant materials or their products [4,6].

Currently, aflatoxins, trichothecenes, ochratoxin A (OTA), zearalenone (ZON), fumonisins, moniliformin, and patulin are the subject of a large number of studies, due to their frequent occurrence and their severe toxic effects [4,7].

The production of mycotoxins in food and feed depends on the substrate for fungal growth, as well as ecological and environmental factors such as temperature and relative humidity. The conditions of storage and the use of fungicides to prevent/limit the growth of fungi are also very important. The interrelations between all mentioned factors, however, are not yet well understood, and production of mycotoxins cannot reasonably be predicted [4].

Among the methods of analysing mycotoxins in food and agricultural products, the most popular are immunochemical-based and chromatographic-based [5,6]. The combination of liquid chromatography with tandem mass spectrometry (MS/MS) allowed the development of highly selective, sensitive, and accurate methods for the determination of mycotoxin. Currently, the best methods seem to be LC–MS/MS-based multi-mycotoxin methods. This method works well, without the need for complicated and time consuming clean-ups and/or multiple analytical techniques [1,2,8,9]. Multi-mycotoxin methods have been described and validated for a wide range of agricultural products, including fruit, cereals, spices, oil seed, and many others [8,10,11].

The disadvantage of all currently available methods of mycotoxin analysis is the inability to assess the spatial distribution of mycotoxins in the tested material. Many authors have pointed out the problems of spatial variability and heterogeneous distribution of mycotoxin contaminations (resulting from a very small proportion of contaminated grains or kernels containing high mycotoxin concentrations) in the bulk of agricultural products [12,13]. Some studies use statistics including Moran’s I, SADIE, Mantel’s tests, the dispersion index, and geostatistical analysis to describe the distributions of fungus/mycotoxin contamination of different bulk commodities [13,14,15]. Despite the fact, however, that the problem of cereal contamination with mycotoxins has been the subject of numerous studies since 1960, no method has yet been developed that assesses the distribution of mycotoxins on the surface of grains, or other plant material, and the depth of their penetration into the interior.

Mass spectrometry imaging (MSI) is a tool that can assist in the determination of the spatial distribution of mycotoxins on tissues of plant origin. MSI has been used for the visualization and analysis of molecules, over a relatively wide range of molecular weight, in complicated biological systems, and with exceptionally good molecular specificity [16]. Notably, so far MSI has been used only in the non-targeted mode, using full mass scanning MS1 spectrum mode [17].

Matrix-assisted laser desorption/ionisation (MALDI) is the selected technique for the molecular imaging of samples of biological tissue. MALDI MSI has been used with success to analyse the spatial distribution of compounds, including proteins and lipids, within different tissues [18,19]. MALDI, however, suffers from a high background level in the low-mass region (under *m/z* 1000), the sweet spot effect, and low ionisation efficiency for low-polarity compounds. These limitations are overcome by matrix-free, laser desorption–ionisation methods, such as surface-assisted laser desorption ionisation (SALDI), including the ^109^AgNPET LDI method [20,21]. Unfortunately, both the MALDI and SALDI methods are based on commercial high-vacuum instruments, which is problematic for many imaged objects, as they lose water and may warp and/or crack during measurements. Additionally, commercial instruments do not provide any kind of metabolite-protecting, tissue-freezing solutions.

Desorption electrospray ionisation (DESI) is the only ambient ionisation technique that has been developed for the imaging of low-molecular-weight compounds in human tissue [22,23,24]. However, this technique is characterized by a relatively low (to 100 μm) spatial resolution. Likewise, it has a low ionisation efficiency for some molecules, as well as low desorption efficiency for molecules that are strongly bound to surfaces [25]. In addition, the DESI depth of sampling is very shallow, which significantly reduces the amount of sample available for testing in the mass spectrometer. As a result, MS signals can be obtained from extracellular fluids instead of the cell’s interior.

In order to overcome these problems, a custom experimental setup, LARESI MSI, was developed [26]. This solution employs a mid-IR laser, a cooling stage, and a tandem SRM/MRM-based measurement mode. In tandem mass spectrometry (MS/MS or MS^2^), the selectivity for a targeted analyte is enhanced by monitoring an ion fragmentation in selected reaction monitoring (SRM) or multiple reaction monitoring (MRM) mode, which dramatically improves sensitivity despite the lower ion detection efficiency. Moreover, it has a wide dynamic range, is very high speed, and is suitable for quantification [27].

The aim of the study was to assess the spatial distribution of grain metabolites and secondary metabolites of *Aspergillus fumigatus* mould under model conditions on an agar medium and spelt grain, using laser ablation-remote-electrospray ionisation mass spectrometry imaging (LARESI MSI) in selected reaction monitoring mode (SRM).

## 2. Results

Ion images for 11 selected metabolites in agar medium and spelt grains infected by *A. fumigatus* mould were obtained by applying the MSI method. Some of the studied metabolites (amino acids: serine, threonine, lysine, glutamic acid, phenylalanine, and glucose) are characteristic of biological tissues (including plant materials and microorganisms), while others (brevianamide F, fumagillin, fumiquinazoline D, fumigaclavine C, and fumitremorgin C) are not naturally present in the grain; they are compounds produced during mould metabolism. We found these secondary metabolites produced by *Aspergillus fumigatus* cultivated on a mineral medium with the addition of compost extract with a concentration from 326 to 96,940 ng/g mycelium [28].

Notably, in the present studies, each metabolite was targeted based on its MS/MS fragmentation using the SRM measurement mode. The method used did not require any additional sample preparation steps; therefore, no alteration of the sample occurred. The agar medium and grain sections were sampled using IR-laser ablation, and all targeted metabolites were tested from the same event of ablation in each pixel. The ESI ion source-produced ions were analysed on a QqQ-configuration QTRAP mass spectrometer containing a linear ion trap. It should be emphasized that this is the first report presenting LARESI imaging results for fungal plant biological material.

A laser beam with a wavelength of 2.94 μm effectively deposits its energy into the strongest water absorption band present in any hydrated biological material. The main advantage of IR laser over near-UV laser for ablation from tissue is the very shallow penetration of laser light, allowing perfect control over ablation depth. For the presented experimental setup, sampling depth was approximately 10 µm for a single laser pulse and arbitrarily deeper for repeated pulses. Among ambient environment MS methods, the ones that use a mid-IR laser for sampling are the most suitable for the analysis of metabolites within biological tissues [26,29].

**Figure 1:**
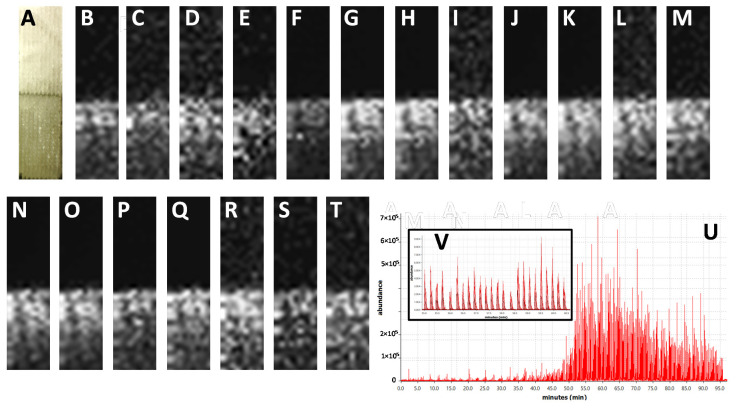
Results of LARESI mass spectrometry imaging of agar samples. A—post-ablation photograph of agar samples without (**A**, upper half) and with *A. fumigatus* (**A**, lower half). (**B**–**T**)—ion images for SRM transitions for *m*/*z* pairs: 284.1/130.1 (**B**), 284.1/103.1 (**C**), 257.1/168.1 (**D**), 257.1/226.1 (**E**), 241.2/154.2 (**F**), 241.2/168.2 (**G**), 459.2/177.1 (**H**), 459.2/131.1 (**I**), 367.1/307.1 (**J**), 367.1/192.1 (**K**), 299.3/167.2 (**L**), 299.3/154.2 (**M**), 444.2/199.1 (**N**), 444.2/171.1 (**O**), 380.3/212.3 (**P**), 380.3/324.3 (**Q**), 403.2/147.1 (**R**), 403.2/130.1 (**S**), 512.3/494.2 (**T**). Panel contains EIC of whole LARESI experiment (**U**), part of EIC panel enlarged for clarity (**V**).

**Figure 2:**
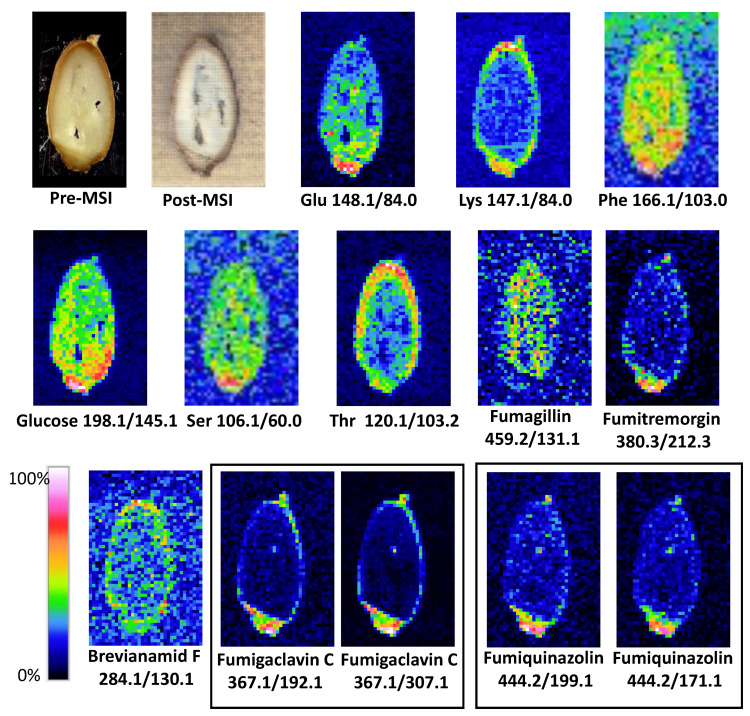
Results of LARESI mass spectrometry imaging of *A. fumigatus* infected spelt. Upper left image is a photograph of pre-ablation section of grain; image on the right is a post-ablation, post-MSI grain section. Other images present ion images generated for SRM pairs stated under each image.

The results of LARESI mass spectrometry imaging of agar samples are presented in Figure 1, while the results obtained for spelt infected with *A. fumigatus* are shown in Figure 2. In the present study, using the LARESI SRM MSI method, images for 11 selected metabolites for *A. fumigatus* are presented. Mass spectrometry parameters for each metabolite in LARESI MSI experiments including *m*/*z* values of the precursor/parent ion and product/daughter ion are presented in Table 1. Moreover, their optimized values for declustering potential, entrance potential, collision energy, and cell exit potential are presented in Table 1. While structures of ions related to SRM/MRM transitions used in this work are shown in Table S1 (Supplementary Material).

The analyses performed (Figure 1) proved that the method is suitable for the detection and localisation of metabolites of microbial origin. Similar measurement conditions were used to investigate the distribution of the studied metabolites in grain. Knowledge about the spatial localisation of mycotoxins should be taken into account when choosing the method of extracting mycotoxins or the method for decontamination of products contaminated with mycotoxins, in order to limit the financial losses of food producers and guarantee consumer safety.

Depending on their type, chemical compounds could be accumulated in the entire volume of the seed, endosperm, seed coat, aleurone layer, or in the embryo. The amino acids glutamic acid, phenylalanine, and serine, as well as monosaccharide glucose, have been shown to be located in the entire spelt volume, with the highest concentrations found in the embryo (Figure 2). A completely different situation occurred in the case of lysine and threonine, which were accumulated in the seed coat (Lys) and the seed coat of the aleurone layer (Thr). It is known from the literature that essential amino acids (arginine, cystine, histidine, isoleucine, leucine, lysine, methionine, phenylalanine, threonine, tryptophan, and valine) are not evenly distributed throughout the wheat grain. It was found that apart from the germ, the highest concentration of essential amino acids occurs in the outer part of the grain [30].

A different spatial distribution in the grain was also found for the metabolites characteristic of mould. Fumigaclavine C, fumitremorgin C, and fumiquinazoline D were mainly located in the embryo and in low concentrations in the seed coat, and they were practically absent in the endosperm of the grain (Figure 2). The research carried out in the present work confirms the earlier observations of mycotoxin content in fractions of processed cereals. The studies published so far showing the concentration of mycotoxins in cereals and their processing products demonstrate that mycotoxins can diffuse deeply into cereal grains. The highest concentrations of mycotoxins are especially found in the fractions of bran and embryos intended for fodder. They are also present in the aleurone layer, and during processing they pass into all mill products such as groats and flours [31].

Sorting and trimming can lower concentrations of mycotoxin because these treatments mechanically remove material contaminated by fungi and their metabolites. These operations, however, do not destroy mycotoxins. Cleaning grains removes kernels with extensive mould growth, ones that are broken, and fine materials, which reduces mycotoxin concentrations [32].

Sydenham et al. (1994) indicated that concentrations of fumonisin in corn were reduced by 26–69% through cleaning alone [33]. This process can also remove scab-infested wheat and barley kernels, which may reduce deoxynivalenol concentrations by 5.5–19% in wheat being prepared for milling [34]. Nevertheless, only 2–3% reduction of ochratoxin A in barley was achieved by cleaning [35]. Park (2002) claimed that physical cleaning, where mould-damaged kernels, seeds, or nuts are removed from the intact commodity, may result in 40–80% reduction of aflatoxins [36]. It should be emphasised that even though sorting, trimming, and cleaning may reduce mycotoxin concentrations in commodities, these operations may not completely remove all contamination. Cleaning efficiency depends on the initial condition of the grain and commodity and the extent of the contamination [32]. The method proposed in the present work may be useful in quickly monitoring the efficiency of cleaning and pre-treatment of cereals, reducing the level of mycotoxins by imaging samples before and after these treatments. It is worth emphasising that since 2005, MSI has been gradually applied in plant research [37,38]. Information on the spatial organisation of proteins and metabolites will greatly improve our understanding of plant metabolism and the biochemical functions of specific plant tissues [39,40,41]. Far less research concerns the spatial distribution of microbial metabolites. Recently, Wisman et al. (2020) used MSI to monitor *A. oryzae* growth during rice koji making (sake brewing process) [42]. Using MSI of glucose, the authors demonstrated that the area of mycelial penetration is directly correlated with the spread of glucose during rice fermentation [42].

The LARESI MSI imaging method was recently employed by Szulc et al. (2020) to detect mycotoxins including ochratoxin B, T2-toxin, aflatoxins B1 and B2, and organic acids on the surfaces of historic photographs [43]. The authors found metabolites that were compatible with the analysis of the fungal biodiversity of tested samples assessed using high-throughput sequencing [43]. Therefore, it is reasonable to use the LARESI MSI imaging method described in the present work for the metabolomic analysis of various plants and agricultural products in real conditions, in situ.

## 3. Conclusions

The presented studies prove the effectiveness of LARESI mass spectrometry imaging in the analysis of the spatial distribution of mycotoxins on the surface of plant material. The biggest advantage of the presented methods is that samples were not modified in any way before the tests, which can be carried out in situ.

The varying distribution of metabolites in spelt grain was demonstrated. Fumigaclavine C, fumitremorgin C, and fumiquinazoline D were located primarily in the embryo, brevianamide F in the seed coat, and fumagillin in the endosperm. The LARESI MSI method can be used in the future for the metabolomic analysis of various plants and agricultural products.

## 4. Materials and Methods

### 4.1. Mould Culture

#### 4.1.1. Culture on Agar Medium

*Aspergillus fumigatus* LOCK CPC 0600 (ITS gene sequences deposited in the National Center for Biotechnology Information GenBank database with no. KC456184) with confirmed toxinogenicity in earlier studies [28] was obtained from the Collection of Pure Culture at the Institute of Fermentation Technology and Microbiology at Lodz University of Technology (LOCK CPC).

The *A. fumigatus* strain was grown on a Petri dish with PDA medium (Potato Dextrose Agar, BTL, Warszawa, Poland) for 10 days at 25 ± 2 °C. Mould cultures on agar medium were used as inoculum for grain infection and were also tested using LARESI SRM MSI.

#### 4.1.2. Grain Contamination

Spelt is gaining popularity as an alternative to wheat because it is ecological and more favourable to human health. Therefore, its grains were selected for research. Commercially available grains of spelt (Melvit, Warszawa, Poland) were used in the study. Ten grams of spelt in 10 mL aqueous glucose solution (glucose 38 g/L) was placed in glass bottles of 100 mL. The grains were allowed to stand at room temperature for 3 days to swell. Then, the samples were autoclaved (15 min, 121 °C), cooled down, and inoculated with 5 mL of spore suspension. Spore suspensions were prepared by adding 10 mL of sterile water (with 0.05% Tween 80) to the agar plate culture of *A. fumigatus*. The number of spores was determined using a Thom cell chamber and confirmed by the culture method. Afterwards, the spore suspensions were diluted to the 10^6^ spores/mL concentration.

The grain cultures were grown in the dark at 25 ± 2 °C for 14 days.

### 4.2. LARESI SRM MSI Experiments

#### 4.2.1. LARESI of Agar Medium Samples

For LARESI SRM MSI agar imaging experiments, around 1 × 1 cm of freshly prepared agar medium (3 mm thickness) was stacked with *A. fumigatus* agar culture on DPA (10 × 10 × 3 mm) on a stainless steel plate (40 × 30 × 0.8 mm, Figure 1). The experiment was performed on a 2 × 8 mm section of stacked object with mould agar placed at the lower half of the object (Figure 1). The resolution used was 10 × 40 pixels, equal to 200 µm X and Y resolution.

#### 4.2.2. LARESI of Spelt Section

Sections of 200 µm thick spelt grain infected by *A. fumigatus* were cut using a cryotome, and the slices (Figure 2, top-left) were mounted on a Peltier stage set to −18 °C to minimise the lateral mixing of compounds in the sample surface. The experiment was performed in a 5.5 × 8.5 mm ablation raster. The resolution used was 35 × 54 pixels, equal to 157 µm X and Y resolution.

For experiments presented in this work, an Nd/YAG-pumped, OPO laser (IR Opolette 2731-HE; Opotek, Carlsbad, CA, USA) producing 4–5 ns pulses with a repetition frequency of 20 Hz set at 2.94 μm wavelength was used. The pulse energy at laser exit aperture was 3.5 mJ (measured using energy meter PE25-SH-V2; Ophir-Spiricon, Logan, UT, USA). The MSI experiments were performed in an airtight PMMA chamber, as shown in Nizioł et al. (2020) [26]. The chamber was pressurized with nitrogen to produce a 2 L/min gas stream entering and leaving the chamber. The sample was placed on a 50 × 50 mm sample stage built on a Peltier cooling plate providing temperature of –18 °C. The sample stage was mounted on a motorised XY-stage (MTS50-Z8; Thorlabs, Newton, NJ, USA). The laser pulses from the OPO laser entered the sample chamber through a 1 inch Infrasil window (Thorlabs, Newton, NJ, USA) and were reflected towards the sample stage by a gold-plated mirror (Thorlabs, Sweden). The beam was focused onto the sample using a 40 mm focal length CaF2 spherical lens (Thorlabs, Sweden), mounted on a Z-axis stage (Thorlabs, Sweden). The laser focus size was 60 ± 10 μm, and the pulse energy measured at the sample surface was approximately 2 mJ. A funnel-type device [26] connected to a 4 mm I.D./6 mm O.D. PTFE tube was positioned over the laser ablation site, allowing laser ablation plume to be transported to the electrospray ionisation (ESI) source of the SCIEX QTRAP 5500 mass spectrometer. An HPLC pump (Agilent G1312A) pumped solvent mixture 2:1 IPA:water with 0.5% acetic acid at 50 μL/min to the electrospray needle. Each pixel was exposed to the laser for 2 s, at a laser pulse repetition rate of 15 Hz. The control and analysis software was described recently [26].

#### 4.2.3. Mass Spectrometer Parameters

A SCIEX QTRAP 5500 mass spectrometer was operated in positive ion mode with selected reaction monitoring (SRM) measurement mode with Q1/Q3/DP/EP/CE and CXP settings, as presented in Table 1.

The settings of the ESI source were as follows: source temperature 500 °C, curtain gas 20 psi, ion source gas 1–30 psi, ion source gas 2–20 psi, ion-spray voltage +5500 V, and collision gas (nitrogen) medium.

## Figures and Tables

**Table 1:** Mass spectrometry parameters in LARESI MSI experiments.

No	Compound Name	Q1 [*m/z*]	Q3 [*m/z*]	Scan Time [ms]	DP [V]	EP [V]	CE [V]	CXP [V]
1	Serine	106.1	60.0	30	6	10	16	7
2	Threonine	120.1	102.1	30	20	14	27	10
3	Lysine	147.1	84.0	30	15	14	23	10
4	Glutamic acid	148.1	84.0	30	21	14	21	10
5	Phenylalanine	166.1	103.0	30	11	14	37	12
6	Glucose	198.1	145.1	30	90	10	20	10
7	Brevianamide F	284.1	130.1	20	86	10	31	8
8	Fumagillin	459.2	131.1	20	101	10	43	22
9	Fumiquinazolin D	444.2	171.1	20	71	10	49	10
10		444.2	199.1		71	10	25	10
11	Fumigaclavin C	367.1	192.1	20	91	10	51	10
12		367.1	307.1		91	10	33	10
	Fumitremorgin C	380.3	212.3	20	91	10	45	12

DP—declustering potential; EP—entrance potential; CE—collision energy; CXP—cell exit potential.

## Supplementary Materials

The following are available online at https://www.mdpi.com/2072-6651/12/11/720/s1, Table S1: Structures of ions related to SRM/MRM transitions used in this work.

## References

[1] Sulyok M., Berthiller F., Krska R., Schuhmacher R. (2006). Development and validation of a liquid chromatography/tandem mass spectrometric method for the determination of 39 mycotoxins in wheat and maize. Rapid Commun. Mass Spectrom..

[2] Capriotti A.L., Caruso G., Cavaliere C., Foglia P., Samperi R., Laganà A. (2011). Multiclass mycotoxin analysis in food, environmental and biological matrices with chromatography/mass spectrometry. Mass Spectrom. Rev..

[3] Bennett J.W., Klich M. (2003). Mycotoxins. Clin. Microbiol. Rev..

[4] Zöllner P., Mayer-Helm B. (2006). Trace mycotoxin analysis in complex biological and food matrices by liquid chromatography–atmospheric pressure ionisation mass spectrometry. J. Chromatogr. A.

[5] Shanakhat H., Sorrentino A., Raiola A., Romano A., Masi P., Cavella S. (2018). Current methods for mycotoxins analysis and innovative strategies for their reduction in cereals: An overview. J. Sci. Food Agric..

[6] Anfossi L., Giovannoli C., Baggiani C. (2016). Mycotoxin detection. Curr. Opin. Biotechnol..

[7] Hussein H.S., Brasel J.M. (2001). Toxicity, metabolism, and impact of mycotoxins on humans and animals. Toxicology.

[8] Sulyok M., Krska R., Schuhmacher R. (2007). A liquid chromatography/tandem mass spectrometric multi-mycotoxin method for the quantification of 87 analytes and its application to semi-quantitative screening of moldy food samples. Anal. Bioanal. Chem..

[9] Malachová A., Sulyok M., Beltrán E., Berthiller F., Krska R. (2014). Optimization and validation of a quantitative liquid chromatography–tandem mass spectrometric method covering 295 bacterial and fungal metabolites including all regulated mycotoxins in four model food matrices. J. Chromatogr. A.

[10] Lacina O., Zachariasova M., Urbanova J., Vaclavikova M., Cajka T., Hajslova J. (2012). Critical assessment of extraction methods for the simultaneous determination of pesticide residues and mycotoxins in fruits, cereals, spices and oil seeds employing ultra-high performance liquid chromatography–tandem mass spectrometry. J. Chromatogr. A.

[11] Beltrán E., Sirtori C., Portolés T., Ripollés C., Sancho J.V., Yusà V., Marín S., Hernández F. (2013). Development of sensitive and rapid analytical methodology for food analysis of 18 mycotoxins included in a total diet study. Anal. Chim. Acta.

[12] Stroka J., Spanjer M., Buechler S., Barel S., Kos G., Anklam E. (2004). Novel sampling methods for the analysis of mycotoxins and the combination with spectroscopic methods for the rapid evaluation of deoxynivalenol contamination. Toxicol. Lett..

[13] Casado M.R., Hubatova M., Weightman R., Magan N., Origgi S. (2009). Geostatistical analysis of the spatial distribution of mycotoxin concentration in bulk cereals. Food Addit. Contam. Part A.

[14] Schmale D.G., Shah D.A., Bergstrom G.C. (2005). Spatial Patterns of Viable Spore Deposition of Gibberella zeae in Wheat Fields. Phytopathology.

[15] Wilhelm K.P., Jones R.K. (2005). Meso- and Microscale Patterns of Fusarium Head Blight in Spring Wheat Fields in Minnesota. Plant Dis..

[16] Caprioli R.M., Farmer T.B., Gile J. (1997). Molecular Imaging of Biological Samples: Localization of Peptides and Proteins Using MALDI-TOF MS. Anal. Chem..

[17] Seeley E.H., Oppenheimer S.R., Mi D., Chaurand P., Caprioli R.M. (2008). Enhancement of protein sensitivity for MALDI imaging mass spectrometry after chemical treatment of tissue sections. J. Am. Soc. Mass Spectrom..

[18] Lippincott-Schwartz J., Snapp E.L., Kenworthy A.K. (2001). Studying protein dynamics in living cells. Nat. Rev. Mol. Cell Biol..

[19] Hájek R., Lísa M., Khalikova M., Jirásko R., Cífková E., Študent V., Vrána D., Opálka L., Vávrová K., Matzenauer M. (2018). HILIC/ESI-MS determination of gangliosides and other polar lipid classes in renal cell carcinoma and surrounding normal tissues. Anal. Bioanal. Chem..

[20] Nizioł J., Rode W., Laskowska B., Ruman T. (2013). Novel Monoisotopic 109AgNPET for Laser Desorption/Ionization Mass Spectrometry. Anal. Chem..

[21] Nizioł J., Ossoliński K., Ossoliński T., Ossolińska A., Bonifay V., Sekuła J., Dobrowolski Z., Sunner J., Beech I.B., Ruman T. (2016). Surface-Transfer Mass Spectrometry Imaging of Renal Tissue on Gold Nanoparticle Enhanced Target. Anal. Chem..

[22] Dill A.L., Eberlin L.S., Zheng C., Costa A., Ifa D.R., Cheng L., Masterson T.A., Koch M.O., Vitek O., Cooks R.G. (2010). Multivariate statistical differentiation of renal cell carcinomas based on lipidomic analysis by ambient ionization imaging mass spectrometry. Anal. Bioanal. Chem..

[23] Alfaro C.M., Jarmusch A.K., Pirro V., Kerian K.S., Masterson T.A., Cheng L., Cooks R.G. (2016). Ambient ionization mass spectrometric analysis of human surgical specimens to distinguish renal cell carcinoma from healthy renal tissue. Anal. Bioanal. Chem..

[24] Tamura K., Horikawa M., Sato S., Miyake H., Setou M. (2019). Discovery of lipid biomarkers correlated with disease progression in clear cell renal cell carcinoma using desorption electrospray ionization imaging mass spectrometry. Oncotarget.

[25] Takáts Z., Wiseman J.M., Gologan B., Cooks R.G. (2004). Mass Spectrometry Sampling Under Ambient Conditions with Desorption Electrospray Ionization. Science.

[26] Nizioł J., Sunner J., Beech I.B., Ossoliński K., Ossolińska A., Ossoliński T., Płaza A., Ruman T. (2020). Localization of Metabolites of Human Kidney Tissue with Infrared Laser-Based Selected Reaction Monitoring Mass Spectrometry Imaging and Silver-109 Nanoparticle-Based Surface Assisted Laser Desorption/Ionization Mass Spectrometry Imaging. Anal. Chem..

[27] Kovarik P., Grivet C., Bourgogne E., Hopfgartner G. (2007). Method development aspects for the quantitation of pharmaceutical compounds in human plasma with a matrix-assisted laser desorption/ionization source in the multiple reaction monitoring mode. Rapid Commun. Mass Spectrom..

[28] Gutarowska B., Skora J., Stępień Ł., Twarużek M., Błajet-Kosicka A., Otlewska A., Grajewski J. (2014). Estimation of fungal contamination and mycotoxin production at workplaces in composting plants, tanneries, archives and libraries. World Mycotoxin J..

[29] Dreisewerd K., Draude F., Kruppe S., Rohlfing A., Berkenkamp S., Pohlentz G. (2007). Molecular Analysis of Native Tissue and Whole Oils by Infrared Laser Mass Spectrometry. Anal. Chem..

[30] Barton-Wright E.C., Moran T. (1946). The microbiological assay of amino acids. II. The distribution of amino acids in the wheat grain. Analyst.

[31] Chełkowski J. (1991). Cereal Grain—Fungi, Mycotoxins and Quality in Drying and Storage.

[32] Bullerman L.B., Bianchini A. (2007). Stability of mycotoxins during food processing. Int. J. Food Microbiol..

[33] Sydenham E.W., Van Der Westhuizen L., Stockenström S., Shephard G.S., Thiel P.G. (1994). Fumonisin-contaminated maise: Physical treatment for the partial decontamination of bulk shipments. Food Addit. Contam..

[34] Abbas H.K., Mirocha C.J., Pawlosky R.J., Pusch D.J. (1985). Effect of cleaning, milling, and baking on deoxynivalenol in wheat. Appl. Environ. Microbiol..

[35] Scudamore K.A., Banks J., Macdonald S.J. (2003). Fate of ochratoxin A in the processing of whole wheat grains during milling and bread production. Food Addit. Contam..

[36] Park D.L., De Vries J.W., Trucksess M.W., Jackson L.S. (2002). Effect of processing on aflatoxin. Mycotoxins and Food Safety.

[37] Imai T., Tanabe K., Kato T., Fukushima K. (2005). Localization of ferruginol, a diterpene phenol, in Cryptomeria japonica heartwood by time-of-flight secondary ion mass spectrometry. Planta.

[38] Mullen A.K., Clench M.R., Crosland S., Sharples K.R. (2005). Determination of agrochemical compounds in soya plants by imaging matrix-assisted laser desorption/ionisation mass spectrometry. Rapid Commun. Mass Spectrom..

[39] Lee Y.J., Perdian D.C., Song Z., Yeung E.S., Nikolau B.J. (2012). Use of mass spectrometry for imaging metabolites in plants. Plant J..

[40] Matros A., Mock H.-P. (2013). Mass Spectrometry Based Imaging Techniques for Spatially Resolved Analysis of Molecules. Front. Plant Sci..

[41] Dong Y., Li B., Malitsky S., Rogachev I., Aharoni A., Kaftan F., Svatoš A., Franceschi P. (2016). Sample Preparation for Mass Spectrometry Imaging of Plant Tissues: A Review. Front. Plant Sci..

[42] Wisman A.P., Tamada Y., Hirohata S., Gomi K., Fukusaki E., Shimma S. (2020). Mapping haze-komi on rice koji grains using β-glucuronidase expressing Aspergillus oryzae and mass spectrometry imaging. J. Biosci. Bioeng..

[43] Szulc J., Ruman T., Karbowska-Berent J., Kozielec T., Gutarowska B. (2020). Analyses of microorganisms and metabolites diversity on historic photographs using innovative methods. J. Cult. Heritage.

