# Supplementary material for: Laser Ablation Remote-Electrospray Ionisation Mass Spectrometry (LARESI MSI) Imaging—New Method for Detection and Spatial Localization of Metabolites and Mycotoxins Produced by Moulds
Source: Toxins (Basel). 2020 Nov 18;12(11):720. doi: 10.3390/toxins12110720 (PMC7698717; doi:10.3390/toxins12110720)
Supplement: Supplementary file 1 [file toxins-12-00720-s001.pdf]

Supplementary Materials: Laser Ablation Remote-Electrospray Ionisation Mass Spectrometry (LARESI MSI) Imaging—New Method for Detection and Spatial Localization of Metabolites and Mycotoxins Produced by Moulds

Justyna Szulc and Tomasz Ruman

Table S1. Structures of ions related to SRM/MRM transitions used in this work.

Compound Name	Q1 [*m/z*] Structure	Q3 [*m/z*] Structure
Serine	106.1 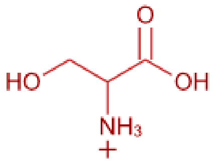	60.0 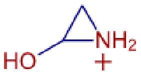
Threonine	120.1 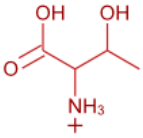	102.1 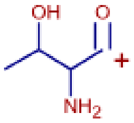
Lysine	147.1 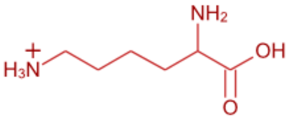	84.0 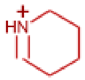
Glutamic acid	148.1 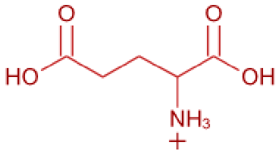	84.0 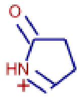
Phenylalanine	166.1 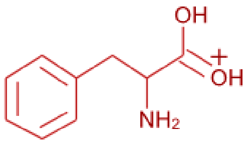	103.0 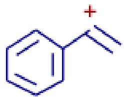
Glucose	198.1 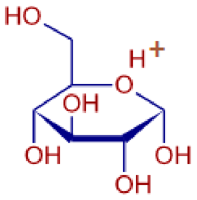	145.1 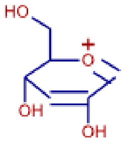
Brevianamide F	284.1	130.1

	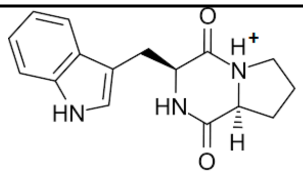	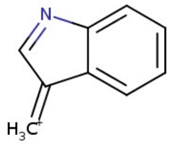
		131.1
Fumagillin	459.2<br>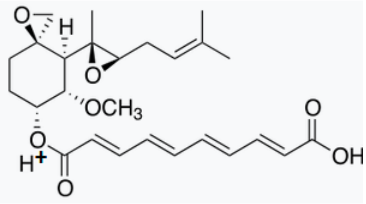	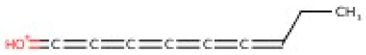
		171.1
Fumiquinazoline D	444.2<br>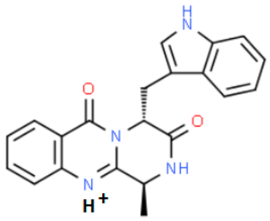	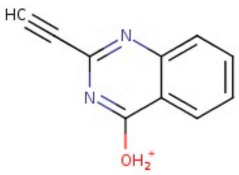
		199.1<br>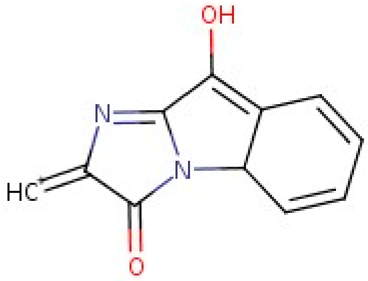
		192.1
Fumigaclavine C	367.1<br>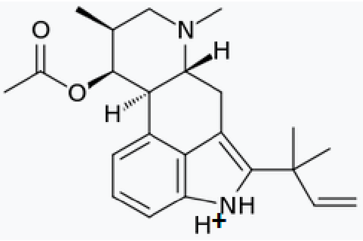	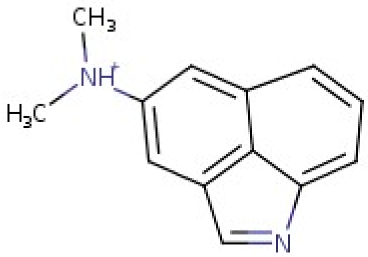
		307.1

		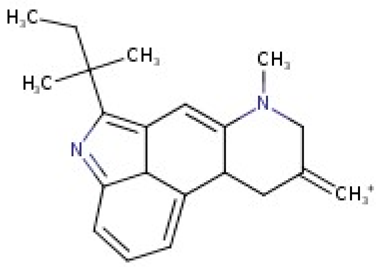
Fumitremorgin C	380.3 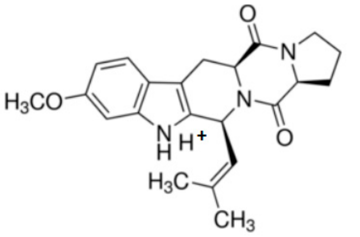	212.3 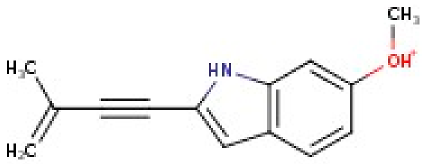
